# Supplementary material for: Triage admission protocol with a centralized quarantine unit for patients after acute care surgery during the COVID-19 pandemic: A tertiary center experience in Taiwan
Source: PLoS One. 2022 Mar 9;17(3):e0263688. doi: 10.1371/journal.pone.0263688 (PMC8906606; doi:10.1371/journal.pone.0263688)
Supplement: Attachment — Submitted filename: Response to reviewers.docx [file pone.0263688.s002.docx]

Comments to the Author

1. Is the manuscript technically sound, and do the data support the conclusions?

The manuscript must describe a technically sound piece of scientific research with data that supports the conclusions. Experiments must have been conducted rigorously, with appropriate controls, replication, and sample sizes. The conclusions must be drawn appropriately based on the data presented.

Reviewer #1: Yes

Reviewer #2: Partly

2. Has the statistical analysis been performed appropriately and rigorously?

Reviewer #1: Yes

Reviewer #2: No

3. Have the authors made all data underlying the findings in their manuscript fully available?

The PLOS Data policy requires authors to make all data underlying the findings described in their manuscript fully available without restriction, with rare exception (please refer to the Data Availability Statement in the manuscript PDF file). The data should be provided as part of the manuscript or its supporting information, or deposited to a public repository. For example, in addition to summary statistics, the data points behind means, medians and variance measures should be available. If there are restrictions on publicly sharing data—e.g. participant privacy or use of data from a third party—those must be specified.

Reviewer #1: Yes

Reviewer #2: No

4. Is the manuscript presented in an intelligible fashion and written in standard English?

PLOS ONE does not copyedit accepted manuscripts, so the language in submitted articles must be clear, correct, and unambiguous. Any typographical or grammatical errors should be corrected at revision, so please note any specific errors here.

Reviewer #1: Yes

Reviewer #2: No

5. Review Comments to the Author

Please use the space provided to explain your answers to the questions above. You may also include additional comments for the author, including concerns about dual publication, research ethics, or publication ethics. (Please upload your review as an attachment if it exceeds 20,000 characters)

Reviewer #1: This study addressed on method of triage admission protocol with a centralized quarantine unit for patients after acute care surgery during the COVID-19 pandemic in one medical center. I have some suggestions listed below.

1. Please correct the mistake of including English-edition highlights.

Thanks for this suggestion. Language and grammar of this manuscript has been edited and corrected by editing company. Please check the editing certification.

2. Taiwan demonstrated the effective control of COVID-19 pandemic. From the experiences in one Taiwan medical center, other countries may learn something from Taiwan. Although this study illustrated one policy of allocation of the patients in need of acute care surgery, the clinical benefit is lacking. In addition to the number of nosocomial infection, I wonder if this novel triage contributes to the less burden of medical professionals or reduction of medical costs.

Further, what is your SOP for the surgical patients with confirmed COVID-19?

- This triage might not reduce burden of medical professionals or medical costs. However, this triage addressed the emergent surgical needs of people in the pandemics. We also ensured a safe hospital environment for medical staffs and other patients.
- COVID-confirmed patients would receive operation in negative pressure, operation room, equipped with isolated ventilation. After operation, the patient would be allocated in COVID-confirmed ward (purple zone) or dedicated ICU where to treat COVID-19 patients.

Reviewer #2: This article described the comprehensive practice handling acute surgery and pre and post OP care during COVID-19 outbreak in the FEMH. The experience was very precious and deserved recording. However, there are many parts that need to be clarified, as followed in detailed:

Major concerns:

1. First of all, the manuscript was not well-organized. It made the reviewer uncomfortable very much to review it.

Thanks for this suggestion. Language and grammar of this manuscript has been edited and corrected by editing company. Please check the editing certification. Comments of the manuscript are removed in the new version of manuscript.

2. The major drawback is no COVID-19 confirmed cases admitted to the unit during the study period. Thus, the study goal, to prevent nosocomial COVID-19 infection was not possible to achieve, despite the authors described in details about the implementation of triage admission protocol and centralized quarantine.

The COVID-19 confirmed cases were admitted to dedicated COVID-confirmed ward (purple zone). We set up the central quarantine unit as a buffer area between the ordinary ward, emergent department (ED), and COVID-confirmed ward. When we were ready to provide more surgical services for patients in ED, most medical care provides were concerned about these patients, for whom more susceptible to COVID-19 infection in pandemics. Medical care providers could prepare themselves and learned the protocol utilizing in the central quarantine unit, and this practice became the new normal after pandemics. Therefore, we might not observe the benefit directly from a number that we stopped nosocomial infection. If another contagious surge develops again, implementation of triage admission protocol and centralized quarantine unit helps us to set up new mindset and practice method which prevents nosocomial infection in future.

3. It’s suggested to describe the scale, involvement and duration of nosocomial outbreak in FEMH. Did the outbreak continue during the study periods?

The scale of the nosocomial outbreak included 27 confirmed cases (12 patients, 12 caregivers, and 3 nurses). 2 ward units were involved, including 88 (52+36) isolation beds. The nosocomial outbreak started on May 14, and no further transmission events or infection cases were attributed to the outbreak. Despite the disease outbreak in FEMH being under control during our study period, our associated preventions are still proceeding. These details is updated in the manuscript.

4. The mean waiting time in ED was longer in phase 1 than phase 2 (397 vs. 532 minutes, p < 0.0001). Although the authors explained that’s positively correlated with the number of surgical patients visiting, it’s suggested to explain more for the triage admission policy. Moreover, there was two different screening methods employed, i.e., RT-PCR, and rapid Ag test + Liat PCR test, respectively. Above 2 methods could have different turn around time. Furthermore, the mean numbers of staff members could be different in these 2 periods, too. The authors may try to put more variables in to analysis.

For RT-PCR, a patch of specimen needed turnaround time about 3.5 hours in our hospital during the pandemic. If a patient presented in ER because of acute surgical disease, we could have reliable diagnosis of COVID-19 in a hour with the help of rapid Ag test and Liat PCR test. Emergent department operated with limited and dynamic medical staffs in these two periods, and we were unable to get actual staff numbers in ER retrospectively.

5. In the table 1, the authors described mean ICU stay was 3 days in both groups Please clarify the zone color of ICU in FEMH according to the triage admission policy. Did the medical teams continue the ICU care? What is the level of PPE ICU?

Critical surgical patients would be transferred to ordinary ICU (green zone) or dedicated ICU (purple zone), depending on whether they were be diagnosed with COVID-19 or not. We encouraged surgeons to communicate with intensivists on phone, and the intensivists in ICU would manage patients’ condition primarily. The PPE regulation maintained same in ICU. For example, we strongly recommended that all medical staff wear basic personal protective equipment (PPE) in the green zones. If working in the purple zones or performing any aerosol-generating procedures, medical staff were required upgrade to advanced PPE

6. The authors did not analyze the needed staff numbers for implementing triage admission and centralized quarantine.

The team members included 3 nurse practitioners and 4 residents to execute the protocol, and 6 attending physicians in different surgical subspecialities enrolled as daily team leader alternately.

7. The authors mentioned that hired foreign caregivers management is a key factor in preventing hospital transmission. Above view point was not relevant to the study results.

Thanks for your suggestion, and this statement is removed from manuscript.

Other suggestions:

1. In Background part of Abstract, line 27-31. Suggest briefly describe why need to establish the system? For example: no space for surgery during pandemic period? Or patients receiving surgery had higher risk of COVID19 infection than before?

2. In Material and Method part of Abstract, line 33-39. Suggest briefly describe the composition of this system.

3. In Result part of Abstract, line 46-47. “The duration of ED stay and waiting time for acute care surgery were longer in Phase II (397 vs. 532 minutes, p < 0.0001)”: Does it mean the duration of ED stay and waiting time were worsen after the system? Suggest show the advantage of this system in the Result part of Abstract.

4. In Conclusion part of Abstract, line 52-57. Suggest briefly describe the advantage of this system.

Thanks for your suggestion, and the abstract has been modified according to your suggestion.

5. In Introduction part, line 72-79. “after community transmission of COVID-19…online video or telephone calls.”. Is this part so-called “Triage admission protocol with a centralized quarantine unit”? If YES, these sentences should be put in the Result part. If NO, suggest delete these sentences since they had no correlation with the title.

Thanks for your suggestion. These contents were re-arranged in new version of
manuscript.

6. In Introduction part, line 80-94. Suggest revise these sentences to focus on the frequent delay of surgery during pandemic period and the drawback of delay of surgery.

Thanks for this suggestion. These contents were re-arranged in new version of
manuscript.

7. In Introduction part, line 100-104, Is there any reference for this system?

8. In Method part, line 132-134, the wards were classified into four parts, is there any reference for this classification? And who decided how to classify these wards?

Inspired by studies by Wake et al[7], Infection Control and Special Response Committee of FEMH classified the wards in the FEMH into four zones: general wards, green zone; COVID assessment ward, yellow zone; COVID alert, red zone; and COVID, purple zone.

9. In Method part, line 139, about the COVID care unit, please describe the member of this unit.

All RT-PCR-positive patients were placed in the COVID-confirmed ward (purple zone), and the patients were cared by dedicated nurses and internists who specialized in infectious disease and chest medicine.

10. In Method part, line 140, about the medical team, please describe the member of this team.

The team members included 3 nurse practitioners and 4 residents to execute the protocol, and 6 attending physicians in different surgical subspecialities enrolled as daily team leader alternately.

11. In Method part, line 147-148. What is the relationship between “reliability of the triage system” with “timely and accurate laboratory tests”?

Our triage admission protocol relied on VTrust rapid test and Cobas® Liat® PCR System. Both tests provided accurate results (VTrust rapid test has 93.1% positive predictive agreement and 99.6% negative predictive agreement. Cobas® Liat® PCR System has 100% positive predictive agreement and 97.4% negative predictive agreement) within 30 minutes.

12. In Method part, line 156-236 “Outpatients who were….. decrease nosocomial COVID-19 transmission..” The authors should describe which part is the regulation of the Taiwan government? Which part consult the reference? And which part is the novel innovation of the hospital?

Inspired by studies by Wake et al, The entrance and exit criteria of green, yellow, and red zone were innovated by Infection Control and Special Response Committee of FEMH. The COVID-confirmed patients were quitted isolation when they met all of the following criteria announced by Taiwan CDC.

13. In Table 1. Suggest to provide P-value for Age, Gender, Surgery department, and ASA classification.

14. There should be reference and brief description of ASA classification in Method part and Table 1.

Thanks for the comment, and manuscript was modified according to these suggestion.

15. In Result part, line 301-331. The authors should try to show the advantage of this “triage admission protocol”. Is there any part of Phase II better than Phase I ?

16. In Discussion part, line 335-338. “The triage admission protocol……and neurologic surgeries.” Are these conclusions proper according to the result of this study? For example, how to prove “The triage admission protocol and centralized quarantine unit secured the hospital”?

Thanks for the comment. The advantage of the triage admission protocol and centralized quarantine unit has been re-written in result part.

17. In Discussion part, line 363-366 “Caregivers form the…. hospitals.” Please cite the reference.

Please see reference [17] Passarelli VC, Faico-Filho K, Moreira LVL, Cunha AP, Carvalho JMA, Barbosa GR, et al*.* Asymptomatic COVID-19 in hospital visitors: Underestimated potential of viral shedding. Int J Infect Dis. 2020;102: 412–414.

18. Table 3, Line 375, “Data are adapted from a webinar by Da-Cheng Qu, Taipei Municipal” Is there any copy-right issue?

I has contacted Dr. Da-Cheng Qu and he agreed the citation.

19. Is Discussion part, line 378-405. Please compare the advantage and disadvantage between these references and the result of this study.

Thanks for your recommendation. The comparison has been added to the manuscript.

20. In Discussion part, line 406-444. Is there any correlation between these discussions with the result of this study? If No, may consider delete these sentences.
