# Supplementary material for: Triage admission protocol with a centralized quarantine unit for patients after acute care surgery during the COVID-19 pandemic: A tertiary center experience in Taiwan
Source: PLoS One. 2022 Mar 9;17(3):e0263688. doi: 10.1371/journal.pone.0263688 (PMC8906606; doi:10.1371/journal.pone.0263688)
Supplement: Attachment — Submitted filename: Response to Reviewers.docx [file pone.0263688.s003.docx]

**Comments to the Author**

1. If the authors have adequately addressed your comments raised in a previous round of review and you feel that this manuscript is now acceptable for publication, you may indicate that here to bypass the “Comments to the Author” section, enter your conflict of interest statement in the “Confidential to Editor” section, and submit your "Accept" recommendation.

Reviewer #1: All comments have been addressed

Reviewer #2: All comments have been addressed

2. Is the manuscript technically sound, and do the data support the conclusions?

The manuscript must describe a technically sound piece of scientific research with data that supports the conclusions. Experiments must have been conducted rigorously, with appropriate controls, replication, and sample sizes. The conclusions must be drawn appropriately based on the data presented.

Reviewer #1: Yes

Reviewer #2: Partly

3. Has the statistical analysis been performed appropriately and rigorously?

Reviewer #1: Yes

Reviewer #2: N/A

4. Have the authors made all data underlying the findings in their manuscript fully available?

The [PLOS Data policy](http://www.plosone.org/static/policies.action#sharing) requires authors to make all data underlying the findings described in their manuscript fully available without restriction, with rare exception (please refer to the Data Availability Statement in the manuscript PDF file). The data should be provided as part of the manuscript or its supporting information, or deposited to a public repository. For example, in addition to summary statistics, the data points behind means, medians and variance measures should be available. If there are restrictions on publicly sharing data—e.g. participant privacy or use of data from a third party—those must be specified.

Reviewer #1: Yes

Reviewer #2: Yes

5. Is the manuscript presented in an intelligible fashion and written in standard English?

PLOS ONE does not copyedit accepted manuscripts, so the language in submitted articles must be clear, correct, and unambiguous. Any typographical or grammatical errors should be corrected at revision, so please note any specific errors here.

Reviewer #1: Yes

Reviewer #2: Yes

6. Review Comments to the Author

Please use the space provided to explain your answers to the questions above. You may also include additional comments for the author, including concerns about dual publication, research ethics, or publication ethics. (Please upload your review as an attachment if it exceeds 20,000 characters)

Reviewer #1: (No Response)

Reviewer #2: Thanks for the dedicated reply from the authors of the article ” Triage admission protocol with a centralized quarantine unit for patients after acute care surgery during the COVID-19 pandemic: A tertiary center experience in Taiwan”

Still, there are some suggestions the authors need to further address and revise in this manuscript, as followed:

1. The authors described the disadvantages of the longer duration of ED stay and waiting time for acute care surgery in Phase II, and correlated the phenomenon to the number of surgical patients visiting the ED and the occupancy ratio in the centralized quarantine unit in the Results. However, no further discussion about the true cause of long ED stay was addressed in the Discussions. Finally in the Conclusions, the authors stated the efficiency was related to the number of medical staff dedicated to the centralized quarantine unit. Please revise and make a logical statement according to the points we emphasize above.

Related discussion was already addressed in line 427-449. During the pandemics, surgical patients from ED could only be admitted in the centralized quarantine unit. The unit had limited beds and nurses, and its efficacy reached plateau soon as more patients admitted in the unit. Appropriate workload planning for the limited number of nurses who were available for inpatient services was an important part of planning for our centralized quarantine unit. Therefore, we concluded that the efficiency was related to the number of medical staff dedicated to the centralized quarantine unit.
2. Please provide more data regarding the occupancy of purple zone in centralized quarantine unit during phase II period.
The purple zone is not part of the centralized quarantine unit, which only included the
 yellow and red zone.
3. In the abstract, it is suggested that:
(1) Add more in the background about ” Why need the centralized quarantine unit and triage admission protocol?” (line 28-31)
(2) Provide the correct number of “nosocomial COVID-19 infection” in phase I and II. (line 42-50)
(3) Provide more statements about the infection control or clinical significance of the centralized quarantine unit and triage admission protocol, especially which outcome is better after intervention (phase II compared to phase I).

I revised the abstract according to your comments. Thanks for your suggestion.
4. In Line 71-73 “However, after community transmission of……into northern Taiwan.” Please cite the reference.

Thanks for this suggestion. The reference was updated.
5. In the Method part, it is suggested to highlight what is the novel innovation of the protocol in this study?

Thanks for this comment. We think the innovation of our protocol is that suspicious or confirmed COVID-19 patients could also be stepped down to less guarded wards after completing isolation. The protocol also allowed patients to be upgraded or downgraded in different tiers, so that the medical resources could be utilized accordingly.
6. In Line 311-318 “Case 1……. and suspicious pneumonia.” Please clarify what is the significance of these three patients?

The details of these patients were omitted in the revised manuscript.
7. In the Result part, please state more clearly about which outcome are better in phase II than phase I.
We thought the major outcome of our study is zero nosocomial infection and marked increase acute surgical inpatient service in phase II, while patients’ surgical risk classification, median total hospital stay, intensive care unit (ICU) stay, intraoperative blood loss, operation time, and the number of patients requiring postoperative ICU care were similar in both periods.

7. PLOS authors have the option to publish the peer review history of their article ([what does this mean?](https://journals.plos.org/plosone/s/editorial-and-peer-review-process#loc-peer-review-history)). If published, this will include your full peer review and any attached files.


If you choose “no”, your identity will remain anonymous but your review may still be made public.


**Do you want your identity to be public for this peer review?** For information about this choice, including consent withdrawal, please see our [Privacy Policy](https://www.plos.org/privacy-policy).

Reviewer #1: No

Reviewer #2: No

While revising your submission, please upload your figure files to the Preflight Analysis and Conversion Engine (PACE) digital diagnostic tool, <https://pacev2.apexcovantage.com/>. PACE helps ensure that figures meet PLOS requirements. To use PACE, you must first register as a user. Registration is free. Then, login and navigate to the UPLOAD tab, where you will find detailed instructions on how to use the tool. If you encounter any issues or have any questions when using PACE, please email PLOS at [figures@plos.org](mailto:figures@plos.org). Please note that Supporting Information files do not need this step.

*In compliance with data protection regulations, you may request that we remove your personal registration details at any time.*  [*(Remove my information/details)*](https://www.editorialmanager.com/pone/login.asp?a=r)*. Please contact the publication office if you have any questions.*
